# Guidelines for multi-model comparisons of the impact of infectious disease interventions

**DOI:** 10.1186/s12916-019-1403-9

**Published:** 2019-08-19

**Authors:** Saskia den Boon, Mark Jit, Marc Brisson, Graham Medley, Philippe Beutels, Richard White, Stefan Flasche, T. Déirdre Hollingsworth, Tini Garske, Virginia E. Pitzer, Martine Hoogendoorn, Oliver Geffen, Andrew Clark, Jane Kim, Raymond Hutubessy

**Affiliations:** 1 Department of Immunization, Vaccines and Biologicals, World Health Organization, Avenue Appia 20, CH-1211 Geneva 27, Switzerland; 2 Centre for Mathematical Modelling of Infectious Disease, London School of Hygiene and Tropical Medicine, London, UK; 3 Modelling and Economics Unit, Public Health England, London, UK; 4 School of Public Health, University of Hong Kong, Hong Kong, SAR China; 5 Department of Social and Preventive Medicine, Université Laval, Quebec, Canada; 6 Centre for Health Economics Research & Modelling Infectious Diseases, Vaccine & Infectious Disease Institute, University of Antwerp, Antwerp, Belgium; 7 Department of Infectious Disease Epidemiology, London School of Hygiene and Tropical Medicine, London, UK; 8 TB Modelling Group, London School of Hygiene and Tropical Medicine, London, UK; 9 Big Data Institute, Li Ka Shing Centre for Health Information and Discovery, University of Oxford, Oxford, UK; 10 Faculty of Medicine, School of Public Health, Imperial College London, London, UK; 11 Department of Epidemiology of Microbial Diseases, Yale School of Public Health, Yale University, New Haven, CT 06511 USA; 12 Institute for Medical Technology Assessment (iMTA), Erasmus University Rotterdam, Rotterdam, The Netherlands; 13 Department of Health Services Research and Policy, London School of Hygiene and Tropical Medicine, London, UK; 14 Department of Health Policy and Management, Harvard T. H. Chan School of Public Health, Boston, USA

**Keywords:** Model comparisons, Policy, Decision-making, Impact modelling, Cost-effectiveness, Infectious diseases, Interventions, Mathematical modelling, Harmonisation

## Abstract

**Background:**

Despite the increasing popularity of multi-model comparison studies and their ability to inform policy recommendations, clear guidance on how to conduct multi-model comparisons is not available. Herein, we present guidelines to provide a structured approach to comparisons of multiple models of interventions against infectious diseases. The primary target audience for these guidelines are researchers carrying out model comparison studies and policy-makers using model comparison studies to inform policy decisions.

**Methods:**

The consensus process used for the development of the guidelines included a systematic review of existing model comparison studies on effectiveness and cost-effectiveness of vaccination, a 2-day meeting and guideline development workshop during which mathematical modellers from different disease areas critically discussed and debated the guideline content and wording, and several rounds of comments on sequential versions of the guidelines by all authors.

**Results:**

The guidelines provide principles for multi-model comparisons, with specific practice statements on what modellers should do for six domains. The guidelines provide explanation and elaboration of the principles and practice statements as well as some examples to illustrate these. The principles are (1) the policy and research question – the model comparison should address a relevant, clearly defined policy question; (2) model identification and selection – the identification and selection of models for inclusion in the model comparison should be transparent and minimise selection bias; (3) harmonisation – standardisation of input data and outputs should be determined by the research question and value of the effort needed for this step; (4) exploring variability – between- and within-model variability and uncertainty should be explored; (5) presenting and pooling results – results should be presented in an appropriate way to support decision-making; and (6) interpretation – results should be interpreted to inform the policy question.

**Conclusion:**

These guidelines should help researchers plan, conduct and report model comparisons of infectious diseases and related interventions in a systematic and structured manner for the purpose of supporting health policy decisions. Adherence to these guidelines will contribute to greater consistency and objectivity in the approach and methods used in multi-model comparisons, and as such improve the quality of modelled evidence for policy.

**Electronic supplementary material:**

The online version of this article (10.1186/s12916-019-1403-9) contains supplementary material, which is available to authorized users.

## Background

### Infectious disease modelling

Health interventions such as vaccines, diagnostics and treatments have greatly reduced suffering and the number of premature deaths due to infectious diseases over the last century. In addition to reducing morbidity and mortality, when highly effective and implemented at a global scale, health interventions can contribute to disease elimination and eradication. However, infectious diseases still account for a substantial disease burden, especially in low-and middle-income countries and, therefore, further optimisation of strategies for their prevention and control are needed.

Mathematical models play a pivotal role in supporting policy decisions about the deployment of health interventions by synthesising evidence about infectious disease epidemiology to provide estimates of the long-term, population-level health impact of interventions [1]. As such, they can inform key questions regarding disease eradication, elimination and control [2]. Health economic modelling can also provide an estimate of economic impact, including risk–benefit, cost-effectiveness, equity and affordability [3]. However, the modelling of infectious diseases has unique complexities related to infectious disease transmission, herd immunity and sources of heterogeneity in rates of infection and disease. These distinct characteristics of infectious diseases mean that disease interventions often have population-level effects that are complex and non-linear. Optimising decisions with regard to interventions, such as the groups to target or the level of coverage to aim for, often requires more analytically complex models, such as transmission dynamic models, that require more behavioural and epidemiological information to parameterise [3].

Uncertainties in infectious disease modelling arise from epidemiological, model parameter and structural uncertainties resulting from the fact that some processes need to be simplified or are not fully understood such as, for example, the natural history of disease (e.g. in the case of many of the neglected tropical diseases and tuberculosis) [2, 4] or people’s behaviour (e.g. sexual activity for sexually transmitted diseases or screening behaviour). Simplifications and shortage of information or data require modellers to make assumptions. Since the various modelling groups can choose different modelling approaches, make distinct assumptions, and use different data to address a decision problem or policy question [5], models can produce disparate results, sometimes leading to different policy recommendations.

### Rationale for multi-model comparisons

According to a recent systematic review of the literature on model comparisons of effectiveness and cost-effectiveness of vaccination, multi-model comparison studies have been performed in the past to synthesise, compare and understand different models’ predictions of the effectiveness and/or cost-effectiveness of an intervention in order to assess model-based evidence for policy-making [6]. More specifically, the systematic review highlighted that model comparison studies have been performed to (1) describe the models that have been used to examine a policy question, (2) better understand the impact of model inputs, model structure, assumptions and parameters on predictions, (3) characterise the robustness of different model predictions to changes in these inputs, structures, assumptions and parameters to assess their impact for policy recommendations, and/or (4) synthesise conclusions from several models in order to inform policy recommendations [6]. Furthermore, the process of going through a model comparison yields other benefits, including improved collaboration between different modelling groups and accelerated spread of technical knowledge to both the modelling community and to non-modellers. It may also enhance the identification of data gaps, guidance of future research, and communication between modellers and decision-makers. Multi-model comparisons may thus have the potential to provide the highest standard of evidence as well as a more rigorous, relevant and unbiased synthesis of current knowledge obtained by mathematical modelling. However, there are also disadvantages of multi-model comparisons; they involve additional time and resources that could be used to model new scenarios and there is also the possibility that independent groups will converge their model parameters and assumptions following comparison and discussion with other groups, such that the models are no longer totally independent.

### Rationale for the multi-model comparison guidelines

Multi-model comparison studies are becoming increasingly popular. For the field of vaccination alone, 115 multi-model comparison studies were identified in a recent systematic review, more than half of which were published since 2011 [6]. Multi-model comparisons have also been performed for non-communicable diseases such as diabetes, cancer and chronic obstructive pulmonary disease [7–9] as well as for questions unrelated to vaccines for infectious diseases [10–15]. However, contrary to other methods of data synthesis, such as systematic reviews and meta-analysis of empirical studies, no clear guidelines exist on how to conduct multi-model comparisons [6]. The guidelines presented herein thus fill a gap and aim to provide a systematic and structured approach to model comparison by presenting principles and practices of model comparisons for interventions against infectious diseases. The guidelines also aim to help researchers improve the completeness and transparency of reporting multi-model comparisons; this should enhance the quality and transparency of multi-model comparison studies and ultimately provide better tools for decision-making.

## Methods

### Process and stakeholders

These guidelines were requested by the Immunization and Vaccines-related Implementation Research Advisory Committee (IVIR-AC) from the World Health Organization (WHO) [16]. IVIR-AC advises WHO on the relevance and applicability of implementation research, including mathematical models and multi-model comparisons for global vaccine recommendations. IVIR-AC recommends multi-model comparisons, rather than single models, to strengthen the evidence underlying policy decisions [17] and has coordinated or commissioned several model comparisons in the past [18–22], some of which have led to changes in WHO recommendations [23–25]. However, these model comparisons were performed on an ad hoc basis, using informal procedures with various methods.

The guideline development process is described below. Full details are described in Additional file 1: Section 1. Briefly, a working group was established, which organised a 2-day meeting with researchers, policy-makers and a journal editor interested in multi-model comparisons. The purpose of the meeting was twofold; first, it served to facilitate discussion on best practices between different disease areas and, second, it was used to obtain consensus on principles and practices for multi-model comparisons (see Additional file 1: Section 2 for the meeting agenda and Additional file 1: Section 3 for the list of presenters and participants). A systematic review of the literature on model comparisons of effectiveness and cost-effectiveness of vaccination provided initial insight into the key pieces of information that had to be included [6]. The model comparison guide was written by the meeting presenters and participants of the workshop, coordinated by the working group.

### Scope

A multi-model comparison is defined as a formal process by which outcomes of two or more mathematical models of health conditions or interventions are used to provide evidence for a decision. We define mathematical models for these guidelines as mechanistic disease models. The outcomes may include immunological (e.g. antibody titres), clinical (e.g. disease episodes and deaths), epidemiological (e.g. disease incidence or force of infection) and/or economic (e.g. cost-effectiveness ratios) endpoints. Generally, multi-model comparisons can be for policy (i.e. to answer a clearly defined policy question by comparing model outcomes for different scenarios) or can have a purely scientific purpose of better understanding the underlying drivers of differences between model predictions. Here, we focus on multi-model comparisons to support policy decisions related to infectious diseases. Hence, we exclude comparisons of models developed purely for better scientific or epidemiological understanding of a disease without an obvious policy question as well as model comparisons performed to better estimate disease burden.

Drolet et al.’s [6] review suggested classifying multi-model comparisons into two categories as (1) comparisons that were based purely on results available in the public domain (e.g. journal articles) and (2) comparisons that were based on generating new model simulations not previously in the public domain. The focus of the present guidelines is on the second category. This document does not provide guidance on how to develop, describe, validate or fit individual models since guidelines already exist for good modelling practice in many disease areas [26, 27].

### Target audience

The primary target audience for these guidelines are researchers carrying out model comparison studies and policy-makers using evidence from model comparison studies to inform policy decisions. The guidelines also target funding agencies, journal editors and reviewers who need to understand the methodology and procedures of model comparisons and must be able to assess the quality of the model comparison.

## Results

The principles we describe here should improve the reliability, transparency and usefulness of multi-model comparisons for decision-making. A summary of the principles and practices, explained in detail below, is provided in Table 1.

**Table 1:** Principles and good practice statements for multi-model comparisons

Principle	Good practice
1. **Policy and research question:** The model comparison should address a relevant, clearly defined policy question	• The policy question should be refined, operationalised and converted into a research question through an iterative process • Process and timelines should be defined in agreement with the policy question
2. **Model identification and selection:** The identification and selection of models for inclusion in the model comparison should be transparent and minimise selection bias	• All models that can (be adapted to) answer the research question should be systematically identified, preferably through a combination of a systematic literature review and open call • Models should be selected using pre-specified inclusion and exclusion criteria, and models identified as potentially suitable but not included should be reported alongside their reason for non-participation • Models used and changes made as part of the comparison process should be well documented • If an internal or external validation was used to limit the model selection, it should be reported
3. **Harmonisation:** Standardisation of input and output data should be determined by the research question and value of the effort needed for this step	• Developing a pre-specified protocol may be useful; if so, it could be published with the comparison results • Modellers should consider fitting models to a common setting or settings • Harmonisation of parameters governing the setting, disease, population and interventions should be considered whilst avoiding changes to fundamental model structures leading to model convergence
4. **Exploring variability:** Between- and within-model variability and uncertainty should be explored	• Multiple scenarios should be explored to understand the drivers of the model results • Sensitivity analysis and what-if analyses (examining extreme scenarios) should be carried out
5. **Presenting and pooling results:** Results should be presented in an appropriate way to support decision-making	• The results for the individual models should be presented, along with within-model uncertainty ranges • Summary measures that combine outcomes of models should only be used if all outcomes support the same policy; it should be clearly communicated whether summary ranges include within-model uncertainty or between-model uncertainty (i.e. the range of point estimates across the model)
6. **Interpretation:** Results should be interpreted to inform the policy question	• Key results and their interpretation to policy questions should be discussed • Key strengths and limitations of the model comparison process and results should be addressed • Key recommendations for next steps should be reported

In the guidelines, it is assumed that there is a coordinator for the model comparison process. This coordinator should ideally be a technically skilled, independent person without a model included in the model comparison exercise; however, for very technical comparisons, someone from one of the participating modelling groups may be needed if there is no independent person with the skills and knowledge to coordinate the exercise.

If those carrying out model comparisons deviate from the guidelines, they should be explicit about this and the reasons for doing so. The guidelines can be adapted for different situations (e.g. to answer a scientific question). Because this is a developing field, the guidelines may need to be updated when new insights or application areas become available.

## Policy and research question

### The model comparison should address a relevant, clearly defined policy question

The focus and the questions that the model comparison is meant to answer should be determined a priori. Policy-makers and/or research funders should be involved at an early stage and throughout the process, and should help define and refine the questions.

Although multi-model comparisons are performed to contribute evidence for policy decisions, they may not necessarily be the best tool for all policy questions. Whether the expected outcome of a multi-model comparison is worth the lengthy resource-intensive process and large time commitment of the involved modelling groups should be carefully considered. To avoid non-participation of modelling groups because of time constraints [22], we recommend dedicated funding for multi-model comparisons, particularly for exercises that assess public health problems in low- and middle-income countries. The role of the funding sources should be explicitly mentioned, as should any potential conflicts of interest.

### Good practice

#### The policy question needs to be refined, operationalised and converted into a research question through an iterative process

A precise and answerable research question is essential for the model comparison process because it shapes the model comparison approach and analysis. The research question directly translates into the scenarios tested, decisions regarding which data to standardise/harmonise and the assumptions made, e.g. regarding setting. Developing a question involves operationalising the policy question into clear scenarios and measurable outputs. Policy-makers’ involvement is essential to ensure that all the important features of the decision problem are correctly represented and that relevant intervention scenarios are tested. In addition, it may be desirable to include disease experts to provide input on the epidemiological and clinical relevance of the policy question.

The original policy question that the model comparison aims to answer should be agreed upon a priori so that the model comparison can be completed in a timely manner to inform policy. If additional policy questions arise, they should only be added if all parties agree that answering these questions is feasible and useful. Occasionally, new or refined research questions arise during the model comparison process; these should be carefully considered in terms of their added value or – if it is a long duration comparison – their overriding importance to provide the most suitable and timely answer to the initial policy question.

#### Process and timelines should be defined in agreement with the policy question

As with any research that aims to be useful for health policy, multi-model comparisons should be organised to provide results at the time they are required for decision-making [28]. Operating within the context of policy decisions frequently requires compromise*.* The need to produce results quickly can reduce the opportunities to systematically identify models, test different scenarios and perform sensitivity analyses to identify sources of variability between models. Furthermore, the time and opportunities for stakeholders to review results, raise questions and refine scenarios may also be minimised. This is acceptable provided that the methodological limitations imposed by fast-tracking are made clear and the limitations and weaknesses of the multi-model comparison are explicitly addressed and/or documented [28]. In some situations, preliminary results may allow decisions to be made.

At the start of a multi-model comparison, it should be clear when the results are needed to support decision-making. A timeframe should be developed to meet that target, including sufficient time for review and revision of scenarios and modelling assumptions. However, developing a timeframe is potentially challenging; often, questions are changed or refined during the model comparison process and the first set of results may lead to decisions to further harmonise the models. These challenges should be anticipated and accommodated to the extent possible in developing timeframes. Furthermore, timelines have to take into account the constraints imposed by the financial resources available for the model comparison exercise as well as by the availability of staff with sufficient expertise to conduct the exercise.

## Model identification and selection

### The identification and selection of models for inclusion in the model comparison should be transparent and minimise selection bias

Once the research question has been determined, a number of models need to be selected for inclusion in the model comparison exercise. The choice of models depends on the policy question; a definition of the types of mathematical model that may be included should be provided, e.g. individual-based models (agent-based models, microsimulation, etc.), compartmental models, Markov models, etc. Defining the mathematical models to be considered for the study will help with the subsequent development of search terms.

The model selection criteria and process should be explicitly defined in advance for transparency. The development of a study protocol for this phase of the model comparison, clearly specifying the search terms, search strategy, and inclusion and exclusion criteria, should be considered as commonly performed for systematic literature reviews. In the study protocol, the decision process for model inclusion should be described, considering, for example, the level of difference between models in order for them to be considered independent, the course of action to be taken when a research group will not or cannot participate, etc. The person or group conducting the selection process should also be explicitly stated regardless of whether they are members of an included modelling group, an external coordinator commissioned for this purpose or the funder/decision-maker.

### Good practice

#### All models that can (be adapted to) answer the research question should be systematically identified, preferably through a combination of a systematic literature review and open call

The combination of a systematic literature review and an open call increases the sensitivity of the search strategy and the likelihood of identifying all eligible models. A systematic literature review is a comprehensive search strategy using explicit search terms and transparent reporting [29] that generally involves a cascading process of searching databases of published studies, conference abstracts, and health technology assessment websites and repositories, reading and analysing the identified studies, and iteratively searching the references of studies captured in the search [30]. Therefore, it has the lowest risk for selection bias. It may also involve actively contacting authors to ask if they know of other models. Instead of conducting a new systematic review, it is also possible to identify models through an existing systematic review if its purpose matches that of the comparison.

Whilst model identification based on a systematic review is a good method to avoid selection bias, this requires time and resources. Furthermore, it may not find models that are not yet adapted for the research question, unpublished or still in development. There are some potential disadvantages of including unpublished models, including lower certainty about quality of models that are not yet peer reviewed and the likelihood that model specifications may still change over the course of the comparison. However, including such models may be particularly important for new policy questions only recently addressed by modellers such as evaluating a health technology in early stages of development. Hence, the systematic review may be supplemented by an open call. An open call means that the purpose and specifics of the model comparison are widely advertised and that everyone who thinks they can fulfil the pre-specified criteria for the model comparison activity in a certain timeframe is encouraged to apply to be part of the model comparison. To achieve its objective, an open call must reach everyone who has developed a model that is potentially eligible and therefore should be widely cast, for example, through advertising on websites or newsletters, announcements at conferences, and active snowballing through potentially relevant groups. However, this process is prone to selection bias because it relies on self-identification of modelling groups and should include an additional quality check for model inclusion.

#### Models should be selected using pre-specified inclusion and exclusion criteria and models identified as potentially suitable but not included should be reported alongside their reason for non-participation

**Fig. 1:**
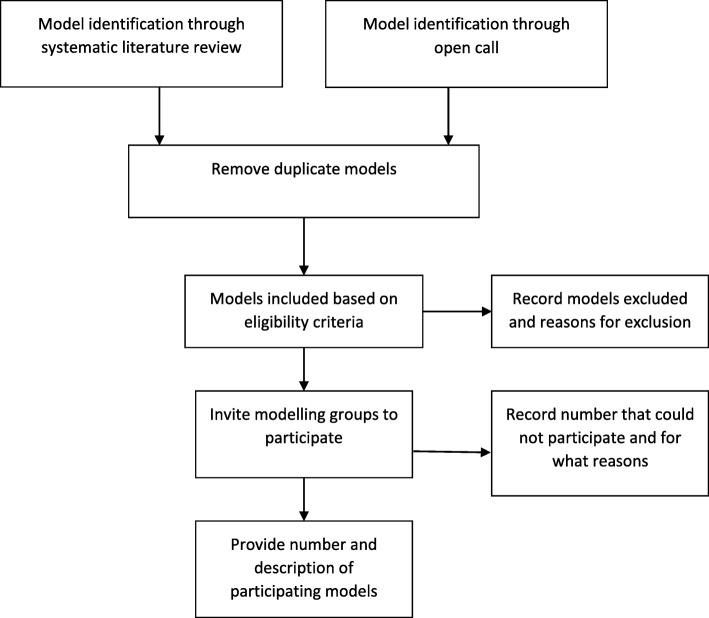
Flow diagram for model identification and inclusion

Transparency requires a clear description of the identified models and the decisions made to select these. Selecting models should, in first instance, be performed using pre-specified selection criteria (e.g. for model characteristics and features) determined by the research question (Fig. 1). Wider inclusion criteria generally introduce greater variability in model assumptions.

An assessment of methodological quality may be performed, for example, by requesting that models are calibrated to epidemiological data and/or that they observe best practice in modelling and adhere to model guidelines such as the Consolidated Health Economic Evaluation Reporting Standards (CHEERS) statement [27]. Models may be excluded if, following data collection, it appears that they cannot be used, they fail to fit (observed) data, or show results that are obviously wrong or biologically or mechanistically impossible and the modellers are not able to correct these errors (Fig. 1). There should be no minimum performance criteria for inclusion in a multi-model comparison, although a subset of models may be selected for a particular analysis provided the reasons for the selection are reported. Models identified as potentially suitable but not included should be reported alongside their reason for non-participation, such as a lack of resources.

Models should be independent, meaning that there must be a qualitative difference in structure or a fundamental difference in the representation or understanding of disease characteristics or transmission networks across the included models. However, even independent models may share components and choices of parameterisation of processes and may rely on similar calibration sources. There is currently no ‘best practice’ approach to characterise model dependence and, often, this will require the coordinator’s valued judgement.

#### Models used and changes made as part of the comparison process should be well documented

Describing the models included in the comparison allows the audience to generally understand how the models differ. It serves to highlight model structures, specific features, data requirements, capabilities and limitations of the models. Models should ideally be described in different ways (e.g. words, diagrams, equations and code) to reduce ambiguity, allow reproducibility, and to facilitate comparisons and information exchange across models. The original model, and any changes made as part of the comparison process, particularly any changes in the structure of the models, should be well documented. Parameter values, their source and their number and type of source data (e.g. epidemiological study, assumption by author, model fit from previous work, etc.) can be described in a table. Details of the models can be provided in a technical annex. If the technical details are long and have been previously described, then they should be either briefly summarised with references to other publications or placed in an online repository.

#### If an internal or external validation was used to limit the model selection, it should be reported

Internal validation evaluates the performance of the model on the population underlying the sample that was used to develop the model. External validation compares the results of the model with the best available evidence [31]. When investigators want to perform an external validation, it is recommended that this is done with data that none of the modelling groups has used for the development and calibration of the model. Often, individual models have already undergone some validation. Additional validation may be performed when new data become available. However, a strong focus on specific performance metrics, in particular if they are based on single datasets, may lead to overconfidence and unjustified convergence, allow compensating errors in models to match certain benchmarks, and may prohibit sufficient diversity of models. On the other hand, discussion and acceptance or rejection of approaches used by individual groups, in order to converge to a meaningful set of common assumptions, is part of the rationale for performing model comparisons, provided that parameters for which there is actually a paucity of evidence remain open to interpretation.

Researchers can develop tools to help the validation process, for example, a standardised format for outputting results, including indicators and ‘sanity checks’ to quickly recognise obvious errors; this also allows for quicker data handling and processing.

## Harmonisation of scenarios, inputs and outputs

### Standardisation of input data and outputs should be determined by the research question and value of the effort needed for this step

To answer a specific policy question, all models simulate the same scenario(s) (e.g. target population, intervention efficacy and coverage) and, possibly, setting (e.g. incidence or prevalence) to determine the extent to which models agree on the health (and economic) impact of the intervention(s) [32]. The rationale for standardising input and output data is that it allows for a more direct comparison of results and conclusions across models, which may otherwise not be possible because models tend to address slightly varying questions, evaluate different scenarios, apply to diverse settings, use differing assumptions regarding other interventions, and report different outcome metrics [32]. By standardising certain assumptions, it is possible to minimise variation in outcomes resulting from such assumptions. Policy-makers and modellers might be interested in exploring the effect of different scenarios, e.g. by varying intervention eligibility, coverage and compliance, or any other assumptions over a pre-specified range or set of values.

Output metrics need to be standardised to enable comparison. The choice of output metrics also depends on the policy question and can include measures of disease incidence or prevalence, costs, disability-adjusted life-years averted and incremental cost-effectiveness ratios, amongst others. It is recommended to use intermediate outputs (e.g. infection and/or disease) in addition to final outputs (e.g. deaths).

### Good practice

#### Developing a pre-specified protocol may be useful; if so, it could be published with the comparison results

Early in the model comparison process, there should be discussion and mutual understanding about the range of mechanisms that are being represented in the models. These should represent what is known (and not known) about the dynamics of disease transmission from person to person, the natural history of disease, the efficacy of available interventions and other fundamentals. Each modelling group should be free to represent and parameterise these processes as they see fit, but an understanding of what is being represented allows for a sharper analysis of differences in outcomes. In addition, the terminology used in the comparison should be defined clearly and in advance (e.g. whether prevalence refers to point, period or lifetime prevalence).

A pre-specified protocol, developed in collaboration with all the modelling groups, describes the analyses that need to be performed and the key outcomes for comparison as well as describing which model elements are standardised; this helps identify the data required by the different modelling groups. The protocol might specify different stages, for example, a first stage in which no changes are made to the models and a second stage with pre-specified changes in the input data. Having a protocol does not preclude further iteration and harmonisation. Initial results might lead to further actions that were not foreseen or specified in the protocol.

#### Modellers should consider fitting models to a common setting or settings

Using data from a common setting to fit models may be useful depending on the research question. For example, for some vector-borne diseases, transmission setting is very important, whilst, for other diseases, the specific setting may be less important provided that the baseline incidence/prevalence is similar. The model comparison can also be carried out for multiple settings, reflecting different disease prevalence or epidemic characteristics [11, 12].

#### Harmonisation of parameters governing the setting, disease, population and interventions should be considered whilst avoiding changes to fundamental model structures leading to model convergence

Model comparisons are conducted in order to understand the differences between models and to examine the impact of these model choices. In order to understand key difference drivers, some parameters governing the setting, disease, population and intervention may need to be harmonised in order to expose the effect of structural differences between models. However, if there are genuine uncertainties around key parameters (such as disease burden or natural history) then it may be better to represent this uncertainty by having the various models calibrated to different parameter sources.

It is important that parameters are not harmonised purely to reduce uncertainty in the range of outcomes predicted by the models, i.e. to make the models converge to a single answer to guide policy [32]. If there are genuine discrepancies between model predictions due to lack of certainty, then these should be reported to decision-makers, who will need to make decisions in the knowledge that there is uncertainty about the outcomes.

There is no generic approach to harmonisation but, generally, it requires the isolation of different areas of uncertainty, e.g. setting, individual parameters and certain elements of structural uncertainty. However, modellers should preferably avoid making changes to fundamental model features such as model structure and/or ‘deep’ parameters such as probability of transmission or duration of natural immunity.

Beyond harmonisation of model parameters, modelling groups may also want to make structural changes to their models in order to better fit data or because of knowledge gained from interaction with other modellers and subject matter experts. Allowing such changes may lead to over-convergence of model outcomes as a result of ‘group think’, so requiring models to retain their original structure (i.e. their published versions or versions at the start of the model comparison process) should be considered. Furthermore, care should be taken that, as a result of any changes made, models do not fall outside the previously set inclusion criteria for model selection and that changes to the models do not result in retrospective widening of the inclusion criteria. If changes are allowed, any changes made during the exercise need to be explicitly documented.

## Exploring variability

### Between- and within-model variability and uncertainty should be explored

**Fig. 2:**
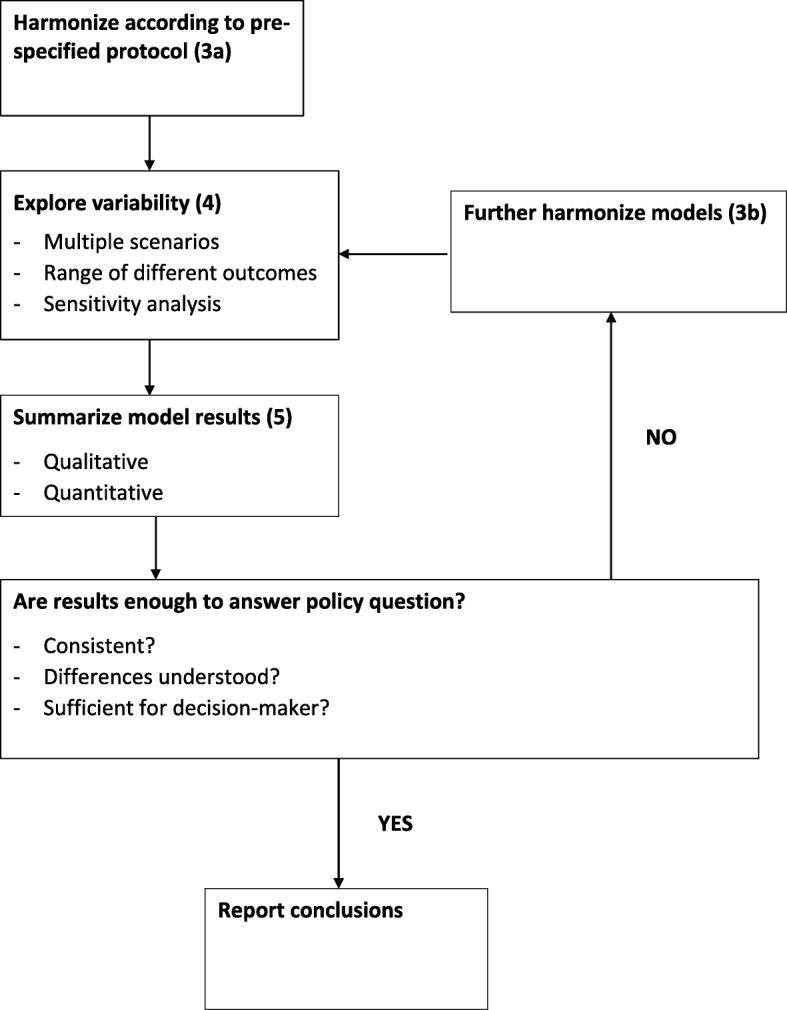
The multi-model harmonisation and comparison process

Exploring between- and within-model variability and uncertainty is a fundamental part of the model comparison. Scenario and sensitivity analyses should be part of the iterative process; the results of these analyses may lead to changes in the models and to refining of the research question (Fig. 2). It may be important to explore different implementation strategies or to evaluate the impact of the intervention in a variety of settings (e.g. with different transmission characteristics) to inform decision-makers.

### Good practice

#### Multiple scenarios should be explored to understand the drivers of the model results

To show the variability between models, a range of different decision scenarios and outcomes should be explored. As is performed for individual models, a base-case scenario can be explored, followed by several different scenarios. These scenarios should include those most relevant to decision-makers but might also reflect different hypotheses, for example, based on epidemiological theory and previous modelling studies, on which model attributes might explain differences in model predictions or outcomes. Scenarios outside of the immediate relevance to the policy decision might allow for a revealing comparison of the impact of important parameters that vary among models. By separately modifying the different aspects of the model (e.g. biological, behavioural and demographic model parameters), it is possible to assess the sensitivity of model output to alternative model structures. This allows modellers to explore and explain the cause of different results [32, 33].

If the model comparison includes many models, it is possible to explore between-model variation and heterogeneity in projected impact using a statistical approach such as a (univariate) meta-regression analysis [32, 33].

#### Sensitivity analysis and what-if analyses (examining extreme scenarios) should be carried out

Presenting the variability between models is one of the core objectives of the model comparison exercise. However, not only between-model heterogeneity should be explored; within-model uncertainty should also be assessed through sensitivity analysis. One possibility is examining extreme scenarios such as a best-case and a worst-case scenario, where all parameters are set at extreme but plausible values that are favourable or unfavourable. Ideally, probabilistic sensitivity analysis should also be performed. Probabilistic sensitivity analysis reflects the combined effects of uncertainty in the value of all the parameters and provides confidence intervals around point estimates (e.g. the base-case results) for each of the models. Performing sensitivity analysis for multiple models allows for the testing of the boundaries of consensus.

## Presenting and pooling results

### Results should be presented in an appropriate way to support decision-making

The results of multi-model comparisons for decision-making should be understandable for the policy-maker. At a minimum, the model results should be described in a qualitative manner, i.e. whether they support or reject a given policy of interest. This is easier when the models are in consensus about the answer to the policy question. If the models do not agree, the modellers should try to explore and explain the underlying causes for these differences. Furthermore, modellers should present the strengths and limitations of the research findings and address the quality of the models included in the comparison. Depending on the research question and the degree of heterogeneity in the model results, the results of multiple models may be pooled to provide one summary estimate plus the variability around that estimate.

### Good practice

#### The results for the individual models should be presented along with within-model uncertainty ranges

Presenting the results of the individual models allows those interpreting and using the findings of the model comparison to assess the variability between the models. Tables, graphs and other visuals can synthesise the large amount of data generated in a model comparison and such visualisations are often preferable to summarising such data in a single pooled estimate.

In addition to the variation between models, the results should show the uncertainty within the different models. The results of (probabilistic) sensitivity analyses can be shown as 95% prediction intervals around the model predictions [31]. However, because the various models may use differing approaches to sensitivity analysis, the reported uncertainty may represent something different for each model. Therefore, uncertainty ranges should never be pooled. Modellers should clearly report the sources of uncertainty for the individual models and the degree to which models have explored uncertainty.

#### Summary measures that combine outcomes of models should only be used if all outcomes support the same policy. It should be clearly communicated whether summary ranges include within-model uncertainty or between-model uncertainty (i.e. the range of point estimates across the model)

Multi-model comparisons often create a large array of outcome data, particularly when many models are included and different scenarios are explored. In such instances, it might be helpful to synthesise model predictions by pooling. In other situations, a specific aim of the model comparison might be to provide some averaged prediction from different models [13, 34, 35]. However, the pooling of results is challenging because models treat uncertainty differently; therefore, this can only be done when models are sufficiently harmonised. If there is a considerable disagreement between the models, modellers should calculate and communicate the uncertainties whilst still providing an accessible message to end-users.

A relatively simple way to summarise results is by majority vote, provided that all the models carry equal weight in terms of reliability. For example, it is possible to show the proportion of posterior estimates across models that supports the decision (e.g. the cost-effectiveness of an intervention). This then includes the uncertainty within each model.

Another summary method is the median, shown together with the variability of the model outcomes, for example, by showing the minimum and maximum estimates and/or posterior values of the models. Such pooled results illustrate the central tendency and variability or robustness of model predictions. In principle, summary statics across models should not be presented or interpreted as sampling distributions for statistical inference such as is done in meta-analyses. Second moments, such as variance or distribution centiles, should probably be avoided. If models are pooled without applying any weights, then implicitly equal weights for all models are assumed. If such an approach is used, modellers should explicitly acknowledge the equal weighting.

A (weighted) mean of model outputs might be a useful method to reduce prediction error for statistical models. For these models, which are data driven, it is possible to obtain model weights from some measure of a model’s ability to simulate observed data (i.e. a model quality metric or index) [13, 36]. However, these methods are still being developed and naïve approaches to weighing, e.g. averaging with equal scores or assigning subjective weights, is discouraged until the underlying science has been developed and proven.

Some model parameter and structure choices are not driven by empirical data but rather by subjective beliefs and values. Examples include the existence (or otherwise) of a biological phenomenon based on theoretical rather than empirical reasoning, and many methodological parameters in health economic models (such as the economic perspective). In this case, the included weights cannot be based on a likelihood measure because there are no data to inform beliefs about the difference between model output and expected population benefits and/or costs. Defining a set of criteria for a model to be ‘credible’ or agreeing on a quality metric is often difficult and there is currently no robust approach for assigning weights to individual model projections. Thus, for many model comparisons, weighting models and/or pooling results is not an appropriate strategy. Instead, it may be better to consider using scenario sensitivity analysis, i.e. presenting results from all models using alternative scenarios.

## Interpretation

### Results should be interpreted to inform the policy question

The results must be interpreted with a focus of maximising the utility of the interpretation to inform the policy question. This should be done simply and clearly. Key results and their meaning to address the policy question should be summarised, followed by a discussion of the key strengths and limitations of the analysis, and, finally, reporting of the next steps. Involving decision-makers during the inception and interpretation phase ensures that the decision-making context is considered and that the results of the model comparison can be used by policy-makers.

### Good practice

#### Key results and their interpretation to policy questions should be discussed

The modellers should provide an unbiased assessment of the modelling evidence and a cautious summary of what they might mean to the decision-maker. If the evidence warrants, a particular strategy should be recommended. To avoid ambiguity, it is encouraged also to report on what results do not mean. Evidence providers should not feel pressured by decision-makers to deliver over-confident or simplistic summaries that hide uncertainty. Mathematical models only address one aspect of a policy question, and the results of a multi-model comparison should be considered together with other evidence such as the feasibility, acceptability and ethical dimensions of a strategy. Thus, the results from a multi-model comparison should be considered as inputs into a decision-making process (such as a health technology assessment) that considers a wider range of relevant criteria.

#### Key strengths and limitations of the model comparison process and results should be addressed

Key strengths and limitations of the analysis should be summarised, with equal emphasis given to both. It may be useful if this could be performed by someone independent of the modelling groups but with input and agreement from all groups. The following items could be addressed: generalisability of results (and/or the consequence of using hypothetical settings), limitations of scenarios explored, assumptions and simplifications made, time and resource constraints that might have limited the analysis, and the selection of included models. Reasons for heterogeneous or divergent results should be explored. Uncertainty should be discussed, even if models give similar results.

It is important to note that agreement between models is not evidence for increased confidence in the results because the selection of models is unlikely to comprehensively explore the entire space of model structure and parameter possibilities. Some features are likely to be shared by many models as a result of the modellers making similar assumptions and simplifications. Agreement between models might also, in part, reflect a level of shared process representation or calibration on particular datasets and does not necessarily imply a higher likelihood of the common answer being ‘correct’.

Researchers should avoid speculation and report on the face validity of results, for example, by evaluating the evidence in line with other considerations.

#### Key recommendations for next steps should be reported

Key recommendations for next steps should be reported and questions that remain unanswered should be highlighted. The researchers should explain what further work is needed to increase certainty, for example, additional data collection and further model analysis. The variation in model results in a multi-model comparison can serve to highlight existing uncertainties and key directions for further data collection such as data shortcomings or lack of information about the natural history of disease [2]. These gaps should be identified and presented to decision-makers in the hope that they will invest in new research and data collection to advance future decision-making.

## Conclusion

These guidelines should help researchers plan, conduct and report model comparisons of infectious diseases and related interventions in a systematic and structured manner for the purpose of supporting health policy decisions. Adherence to these guidelines will contribute to greater consistency and objectivity in the approach and methods used in multi-model comparisons, and as such improve the quality of modelled evidence for policy. Furthermore, we hope that the guidelines will also help policy-makers and others interested in multi-model comparisons to more easily understand and appraise the methodology used as well as the strengths and limitations of multi-model comparisons.

## Additional file

Additional file 1: **Section 1.** Guidelines development process. **Section 2.** Meeting Agenda. **Section 3.** Meeting presenters and/or workshop participants. (DOCX 21 kb)

## Data Availability

Not applicable.

## Abbreviations

IVIR-AC: Immunization and Vaccines-related Implementation Research Advisory Committee
WHO: World Health Organization

## References

[1] Egger M, Johnson L, Althaus C, Schöni A, Salanti G, Low N, Norris S (2018). Developing WHO guidelines: time to formally include evidence from mathematical modelling studies. F1000Res.

[2] Hollingsworth TD, Medley GF (2017). Learning from multi-model comparisons: collaboration leads to insights, but limitations remain. Epidemics..

[3] Jit M, Brisson M (2011). Modelling the epidemiology of infectious diseases for decision analysis: a primer. Pharmacoeconomics..

[4] Vynnycky E, Fine PE (1997). The natural history of tuberculosis: the implications of age-dependent risks of disease and the role of reinfection. Epidemiol Infect.

[5] Kim JJ, Brisson M, Edmunds WJ, Goldie SJ (2008). Modeling cervical cancer prevention in developed countries. Vaccine..

[6] Drolet M, Bénard É, Jit M, Hutubessy R, Brisson M (2018). Model comparisons of the effectiveness and cost-effectiveness of vaccination: a systematic review of the literature. Value Health.

[7] Mount Hood 4 Modeling Group (2007). Computer modelling of diabetes and its complications. A report on the fourth Mount Hood challenge meeting. Diabetes Care.

[8] Berry DA, Cronin KA, Plevritis SK, Fryback DG, Clarke L, Zelen M (2005). Effect of screening and adjuvant therapy on mortality from breast cancer. N Engl J Med.

[9] Hoogendoorn M, Feenstra TL, Asukai Y, Borg S, Hansen RN, Jansson S-A (2014). Cost-effectiveness models for chronic obstructive pulmonary disease: cross-model comparison of hypothetical treatment scenarios. Value Health.

[10] UNAIDS/WHO/SACEMA Expert Group on Modelling the Impact and Cost of Male Circumcision for HIV Prevention (2009). Male circumcision for HIV prevention in high HIV prevalence settings: what can mathematical modelling contribute to informed decision making?. PLoS Med.

[11] Eaton JW, Menzies NA, Stover J, Cambiano V, Chindelevitch L, Cori A (2013). Health benefits, costs, and cost-effectiveness of earlier eligibility for adult antiretroviral therapy and expanded treatment coverage: a combined analysis of 12 mathematical models. Lancet Glob Health.

[12] Houben R, Menzies NA, Sumner T, Huynh GH, Arinaminpathy N, Goldhaber-Fiebert JD (2016). Feasibility of achieving the 2025 WHO global tuberculosis targets in South Africa, China, and India: a combined analysis of 11 mathematical models. Lancet Global Health.

[13] Smith ME, Singh BK, Irvine MA, Stolk WA, Subramanian S, Hollingsworth TD, Michael E (2017). Predicting lymphatic filariasis transmission and elimination dynamics using a multi-model ensemble framework. Epidemics..

[14] Halloran ME, Ferguson NM, Eubank S, Longini IM, Cummings DAT, Lewis B (2008). Modeling targeted layered containment of an influenza pandemic in the United States. Proc Natl Acad Sci U S A.

[15] Althaus CL, Turner KME, Schmid BV, Heijne JCM, Kretzschmar M, Low N (2012). Transmission of *Chlamydia trachomatis* through sexual partnerships: a comparison between three individual-based models and empirical data. J R Soc Interface.

[16] World Health Organization. Immunization and Vaccines Related Implementation Research Advisory Committee (IVIR-AC). http://www.who.int/immunization/research/committees/ivir_ac/en/. Accessed 1 May 2019.

[17] Hutubessy R, Henao AM, Namgyal P, Moorthy V, Hombach J (2011). Results from evaluations of models and cost-effectiveness tools to support introduction decisions for new vaccines need critical appraisal. BMC Med.

[18] Chaiyakunapruk N, Somkrua R, Hutubessy R, Henao AM, Hombach J, Melegaro A (2011). Cost effectiveness of pediatric pneumococcal conjugate vaccines: a comparative assessment of decision-making tools. BMC Med.

[19] Postma MJ, Jit M, Rozenbaum MH, Standaert B, Tu HA, Hutubessy RC (2011). Comparative review of three cost-effectiveness models for rotavirus vaccines in national immunization programs; a generic approach applied to various regions in the world. BMC Med.

[20] Jit M, Demarteau N, Elbasha E, Ginsberg G, Kim J, Praditsitthikorn N (2011). Human papillomavirus vaccine introduction in low-income and middle-income countries: guidance on the use of cost-effectiveness models. BMC Med.

[21] Penny MA, Verity R, Bever CA, Sauboin C, Galactionova K, Flasche S (2016). Public health impact and cost-effectiveness of the RTS,S/AS01 malaria vaccine: a systematic comparison of predictions from four mathematical models. Lancet..

[22] Flasche S, Jit M, Rodriquez-Barraquer I, Coudeville L, Recker M, Koelle K (2016). The long-term safety, public health impact, and cost-effectiveness of routine vaccination with a recombinant, live-attenuated dengue vaccine (Dengvaxia): a model comparison study. PLoS Med.

[23] Malaria vaccine (2016). WHO position paper – January 2016. Wkly Epidemiol Rec.

[24] Human papillomavirus vaccines (2017). WHO position paper, May 2017. Wkly Epidemiol Rec.

[25] Dengue vaccine (2018). WHO position paper – September 2018. Wkly Epidemiol Rec.

[26] Eddy DM, Hollingworth W, Caro JJ, Tsevat J, McDonald KM (2012). Wong JB; ISPOR−SMDM modeling good research practices task force. Model transparency and validation: a report of the ISPOR-SMDM modeling good research practices task Force-7. Value Health.

[27] Husereau D, Drummond M, Petrou S, Greenberg D, Augustovski F, Briggs AH (2013). Consolidated health economic evaluation reporting standards (CHEERS) statement. BMJ..

[28] Whitty CJM (2015). What makes an academic paper useful for health policy?. BMC Med.

[29] Liberati A., Altman D. G, Tetzlaff J., Mulrow C., Gotzsche P. C, Ioannidis J. P A, Clarke M., Devereaux P J, Kleijnen J., Moher D. (2009). The PRISMA statement for reporting systematic reviews and meta-analyses of studies that evaluate healthcare interventions: explanation and elaboration. BMJ.

[30] Higgins JPT, Green S (editors). Cochrane Handbook for Systematic Reviews of Interventions Version 5.1.0 [updated March 2011]. Cochrane Collaboration, 2011. http://handbook.cochrane.org.

[31] Ultsch B, Damm O, Beutels P, Bilcke J, Brüggenjürgen B, Gerber-Grote A (2016). Methods for health economic evaluation of vaccines and immunization decision frameworks: a consensus framework from a European vaccine economics community. Pharmacoeconomics..

[32] Eaton JW, Johnson LF, Salomon JA, Bärnighausen T, Bendavid E, Bershteyn A (2012). HIV treatment as prevention: systematic comparison of mathematical models of the potential impact of antiretroviral therapy on HIV incidence in South Africa. PLoS Med.

[33] Brisson M, Bénard É, Drolet M, Bogaards JA, Baussano L, Vänskä S (2016). Population-level impact, herd immunity, and elimination after human papillomavirus vaccination: a systematic review and meta-analysis of predictions from transmission-dynamic models. Lancet Public Health.

[34] Hoeting JA, Magidan D, Raftery AE, Volinsky CT (1999). Bayesian model averaging: a tutorial. Stat Sci.

[35] Raftery AE, Gneiting T, Balabdaoui F, Polakowski M (2005). Using Bayesian model averaging to calibrate forecast ensembles. Mon Weather Rev.

[36] Park J, Goldstein J, Haran M, Ferrari M (2017). An ensemble approach to predicting the impact of vaccination disease in Niger. Vaccine..

