# Supplementary material for: Guidelines for multi-model comparisons of the impact of infectious disease interventions
Source: BMC Med. 2019 Aug 19;17:163. doi: 10.1186/s12916-019-1403-9 (PMC6699075; doi:10.1186/s12916-019-1403-9)
Supplement: Supplementary file 1 — Section 1. Guidelines development process. Section 2. Meeting Agenda. Section 3. Meeting presenters and/or workshop participants. (DOCX 21 kb) [file 12916_2019_1403_MOESM1_ESM.docx]

**Additional file 1**

**Content**

Section 1: Guidelines development process ………………………………………………………………………. 2

Section 2: Meeting Agenda ……………………………………………………………………………………………….. 4

Section 3: Meeting presenters and/or workshop participants …………………………………………… 5

**Section 1: Guidelines development process**

Development of these guidelines was initiated by IVIR-AC because of the increased role of multi-model comparisons in the development of policy related to vaccines and immunisations. In May 2016, IVIR-AC discussed fundamental principles of good practice for multi-model comparisons. Then, a systematic review of vaccine model comparisons was commissioned to describe the methodologies used and to identify the strengths and limitations of the different approaches [6]. The systematic review provided initial insight into the items to consider for inclusion in the guidelines. In March 2018, a working group was established consisting of members of IVIR-AC, staff and consultants of the WHO Department of Immunization, Vaccines and Biologicals (IVB), and researchers from the Centre for the Mathematical Modelling of Infectious Diseases (CMMID) at the London School of Hygiene and Tropical Medicine (LSHTM). The working group organized a two-day meeting with researchers, policy-makers and a journal editor interested in multi-model comparisons. While the focus of the discussion was on multi-model comparisons in infectious diseases, participants were also drawn from other fields of health research, including cancer, diabetes and chronic obstructive pulmonary disease in order to gain insight from those other fields where model comparisons have been conducted. The meeting was held in London, United Kingdom from 12 to 13 June 2018. The first day of the meeting consisted of an open scientific seminar at which people who carried out multi-model comparisons presented their experiences, particularly focusing on lessons learned. The scientific seminar was recorded and is available through the links provided^[[1]](#footnote-1)^. Relevant pre-reading material was made available to attendants of the scientific seminar [2, 6, 14]. The purpose of this day was to facilitate discussion on best practice, obtaining input from different disease areas. The second day of the meeting was a closed workshop in which the guiding principles and practice statements were developed. Members of the writing group introduced a topic by raising outstanding questions which were then discussed in detail. Key principles and practices were agreed on using a consensus process consisting of iterative discussions. The principles and practice statements were based on the scientific literature and the participants' experience with multi-model comparisons.

The model comparison guidelines were written by the meeting presenters and participants of the workshop, coordinated by the writing committee. The draft guidelines followed four rounds of revisions in which suggestions and comments were incorporated and addressed. Furthermore, the draft guidelines were presented and discussed at the IVIR-AC meeting in France (24-25 September 2018). No external funding was available for the development of the guide; WHO and CMMID, LSHTM jointly facilitated the face-to-face meeting.

**Section 2: Meeting Agenda**

**Tuesday, 12 June 2018 – Scientific Conference**

| **Time** | **Topic** | **Presenter** |
| --- | --- | --- |
| *8.30 – 9.00* | *Registration* |  |
| 9.00 – 9.15 | Welcome | Graham Medley |
| 9.15 – 9.30 | Introduction | Raymond Hutubessy |
| 9.30 – 10.00 | Diabetes, Mount Hood Challenge | Philip Clarke |
| 10.00 – 10.30 | Cancer, CISNET | Jane Kim |
| *10.30 – 11.00* | *Coffee / tea break* |  |
| 11.00 – 11.30 | COPD | Martine Hoogendoorn |
| 11.30 - 12.00 | Tuberculosis | Richard White |
| 12.00 – 12.30 | Sleeping sickness / vector borne diseases | Matt Keeling |
| 12.30 – 12.40 | HIV | Timothy Hallett |
| *12.40 – 13.30* | *Lunch* |  |
| 13.30 – 14.00 | Neglected tropical diseases | Deirdre Hollingsworth |
| 14.00 – 14.15 | WHO IVIR-AC model comparisons | Mark Jit |
| 14.15 – 14.45 | Dengue | Stefan Flasche |
| 14.45 – 15.15 | HPV | Marc Brisson |
| *15.15 - 15.45* | *Coffee / tea break* |  |
| 15.45 – 16.15 | Typhoid | Virginia Pitzer |
| 16.15 – 16.45 | Systematic review of model comparisons | Marc Brisson |
| 16.45 – 17.00 | Model Comparison Guide | Saskia den Boon |
| 17.00 – 17.30 | Closing comments | Graham Medley & Raymond Hutubessy |

**Wednesday, 13 June 2018 – Workshop**

| **Time** | **Topic** | **Presenter** |
| --- | --- | --- |
| 9.00 – 9.45 | Proposed study design for model comparisons | Marc Brisson |
| 9.45 – 10.30 | Search strategy & model selection | Philippe Beutels |
| *10.30-11.00* | *Coffee / tea break* |  |
| 11.00 – 11.45 | Model description & quality assessment | Raymond Hutubessy |
| 11.45 – 12.30 | Standardization of simulation scenarios & model inputs and outputs | Mark Jit |
| *12.30 – 13.30* | *Lunch* |  |
| 13.30 – 14.15 | Data analysis: quantifying variability (pooling results, illustrating uncertainty) | Graham Medley |
| 14.15 – 15.00 | Data analysis: methods to understand heterogeneity in results | Mark Jit |
| *15.00 – 15.30* | *Coffee / tea break* |  |
| 15.30 – 16.00 | Next steps | Saskia den Boon |

**Section 3: Meeting presenters and/or workshop participants**

| Philippe Beutels | University of Antwerp, Belgium |
| --- | --- |
| Saskia den Boon | World Health Organization, Switzerland |
| Philip Clarke | University of Oxford, UK |
| Oliver Geffen | Imperial College London, UK |
| Marc Brisson | Université Laval, Canada |
| Nathorn Chaiyakunapruk | Monash University, Malaysia |
| Andrew Clark | LSHTM, UK |
| John Edmunds | LSHTM, UK |
| Neil Ferguson | Imperial College London, UK |
| Stefan Flasche | LSHTM, UK |
| Timothy Hallett | Imperial College London, UK |
| Deirdre Hollingsworth | University of Oxford, UK |
| Martine Hoogendoorn | Erasmus University, iMTA, the Netherlands |
| Raymond Hutubessy | World Health Organization, Switzerland |
| Tini Garske | Imperial College London, UK |
| Mark Jit | LSHTM, UK |
| Matt Keeling | Warwick University, UK |
| Jane Kim | Harvard University, USA |
| Jeremy Lauer | World Health Organization, Switzerland |
| Graham Medley | CMMID, LSHTM, UK |
| Virginia Pitzer | Yale University, USA |
| Richard White | LSHTM, UK |
|  |  |
| Lin Lee (observer) | BioMed Central |

1. Session 1 - [https://panopto.lshtm.ac.uk/Panopto/Pages/Viewer.aspx?id=57a6cfc5-44a0-4006-a99e-afc32cb6fe4f](https://eur03.safelinks.protection.outlook.com/?url=https%3A%2F%2Fpanopto.lshtm.ac.uk%2FPanopto%2FPages%2FViewer.aspx%3Fid%3D57a6cfc5-44a0-4006-a99e-afc32cb6fe4f&data=02%7C01%7C%7C005e47fbd012423c9bfe08d5cfb8b37d%7C84df9e7fe9f640afb435aaaaaaaaaaaa%7C1%7C0%7C636643314539675278&sdata=el%2FbCZhusw3rELRbcN7jowwjgkv9Uwg7VyC5SLpt5Sg%3D&reserved=0)

   Session 2 - [https://panopto.lshtm.ac.uk/Panopto/Pages/Viewer.aspx?id=757dadfd-9780-42d6-ae28-35f21591cebe](https://eur03.safelinks.protection.outlook.com/?url=https%3A%2F%2Fpanopto.lshtm.ac.uk%2FPanopto%2FPages%2FViewer.aspx%3Fid%3D757dadfd-9780-42d6-ae28-35f21591cebe&data=02%7C01%7C%7C005e47fbd012423c9bfe08d5cfb8b37d%7C84df9e7fe9f640afb435aaaaaaaaaaaa%7C1%7C0%7C636643314539675278&sdata=3phmwZ%2FGN%2BKv3jvrqa9xvSGiQie0WeXzry71fBV7AsQ%3D&reserved=0) [↑](#footnote-ref-1)
